# Non-invasive monitoring survey in Beppu hot spring area using gravity measurements, passive image interferometry, and InSAR

**DOI:** 10.1038/s41598-026-51419-9

**Published:** 2026-05-06

**Authors:** Yohei Morifuji, Hiroaki Sato, Jun Nishijima, Masayuki Kuriyama, Kenji Kubota, Tatsunori Ikeda

**Affiliations:** 1 Sustainable System Research Laboratory, Central Research Institute of Electric Power Industry, 1646 Abiko, Abiko-shi, Chiba 270-1194 Japan; 2 Department of Earth Resources Engineering, Faculty of Engineering, Kyushu University, 744 Motooka Nishi-ku, Fukuoka, 819-0395 Japan

**Keywords:** Hot spring, Reservoir monitoring, Gravity measurement, Passive image interferometry, InSAR, Gas saturation, Natural hazards, Solid Earth sciences

## Abstract

This paper presents the findings of a comprehensive non-invasive monitoring survey of the reservoir in the Beppu hot spring area, which has the largest discharge and the highest average temperature among hot springs in Japan. Interferometric synthetic aperture radar (InSAR) indicates a settlement of 15 mm over the past decade, even in areas with active geothermal use, demonstrating negligible subsidence compared to that in other geothermal areas reported globally. Gravimeters and seismometers were installed at the surface in two locations: one where geothermal manifestations and usage were particularly active and another where there were no discernible geothermal signs. Comparative observations over two and a half years revealed irregular variations in gravity and velocity at the high-geothermal activity site. Seasonal changes in gravity and velocity were observed at another site. The results of each non-invasive monitoring are interpreted as follows: although InSAR detected no significant subsidence, short‑term changes in density and elastic properties capable of producing gravity and velocity variations may have occurred in the subsurface. Based on rock‑physics considerations, we show that, as one possible mechanism, if several percent of gas‑saturation change had occurred in association with production activities, the resulting changes in density and elastic properties could have given rise to the observed changes in gravity and seismic velocity. In hot‑spring regions, production rate and reservoir water‑level data are often unavailable, and in this study too, the lack of such data made it difficult to fully separate the multiple factors that could contribute to gravity and velocity variations. Meanwhile, attempts to capture short‑term subsurface variations through gravity and velocity monitoring may contribute to assessing the risk of overextraction, similarly to long‑term InSAR deformation time‑series analyses.

## Introduction

**Fig. 1:**
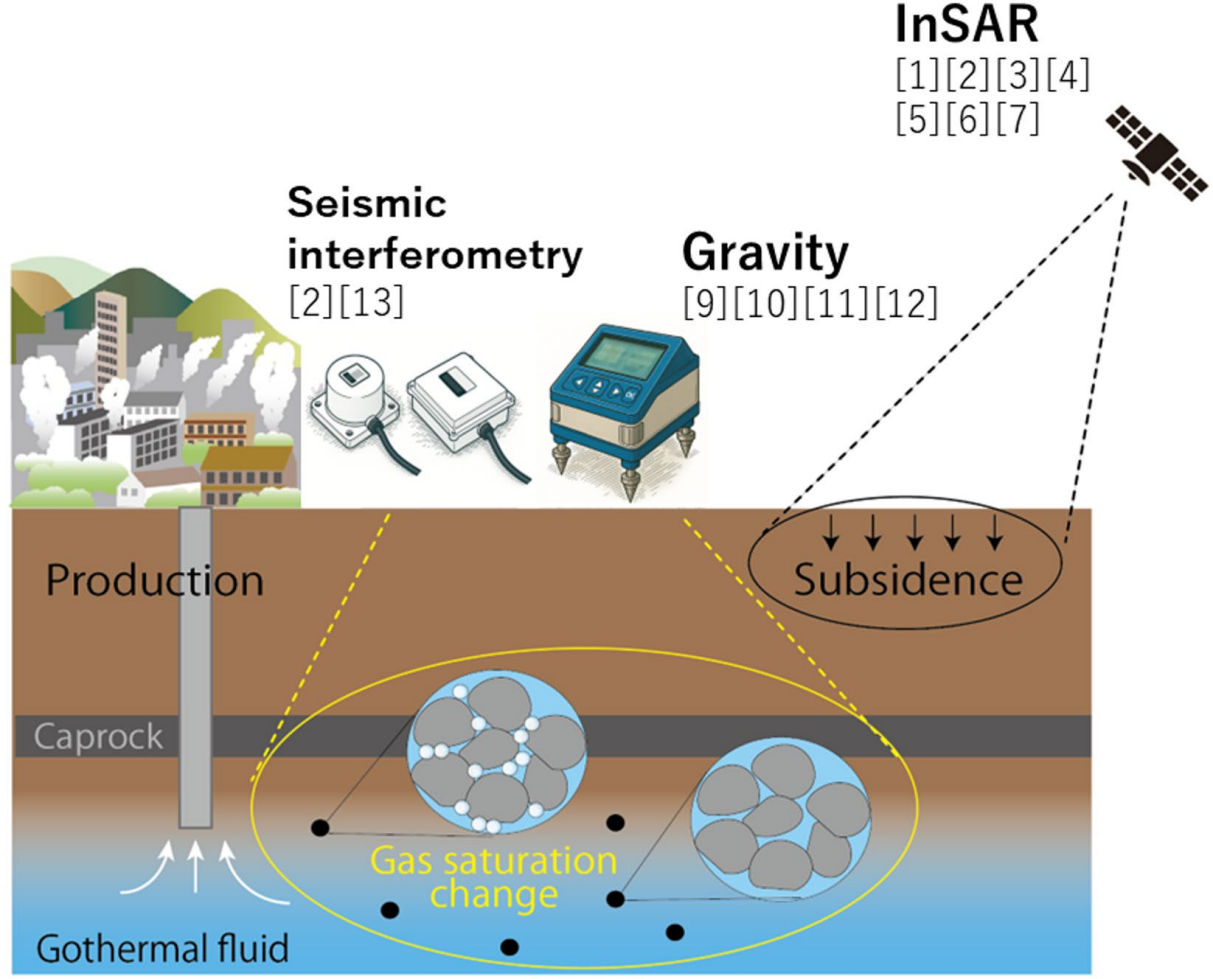
Surface and subsurface changes potentially observable by each non-invasive monitoring method in geothermal areas (synthesized from previous studies^1–7,9–13^).

Geothermal areas can experience subsidence associated with production. Long-term monitoring of surface deformation by ground-based observations and interferometric synthetic aperture radar (InSAR) has been conducted in geothermal areas globally^1–7^. Pressure and saturation changes occur underground at geothermal sites following production^8^. Such changes can be monitored from the surface using non-invasive geophysical methods. Specifically, repeated microgravity measurements^9–12^ and passive image interferometry (PII)^2,13^ can be used to explore pressure and saturation changes. Based on these previous studies, a conceptual illustration summarizing surface and subsurface changes induced by geothermal production, as potentially observed by each monitoring method, is provided in Fig. 1.

**Fig. 2:**
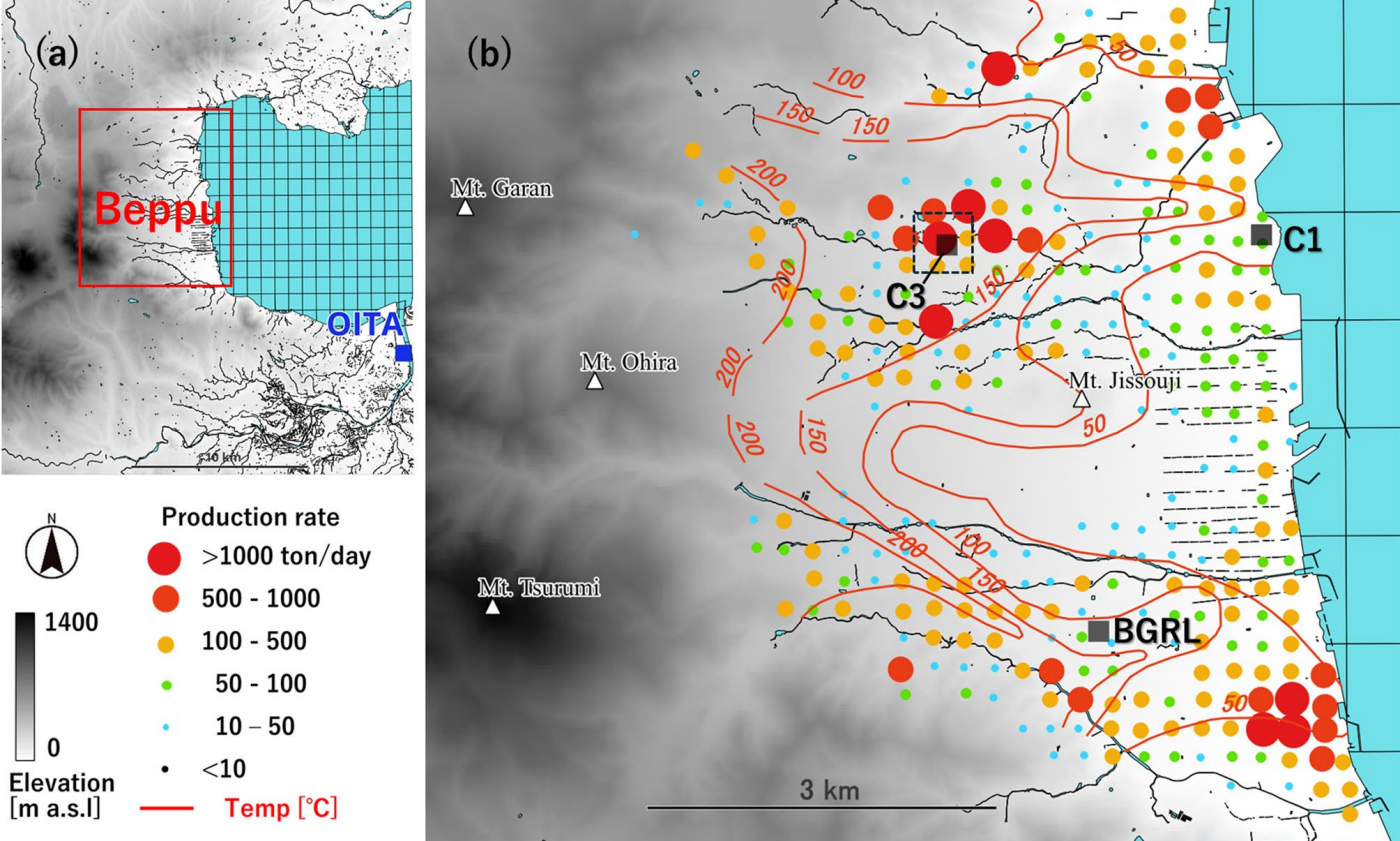
Study area. (a) Beppu hot spring area on Kyushu Island, Japan. Blue square: Automated Meteorological Data Acquisition System (AMeDAS) OITA observation point. The map was created using QGIS (version 3.36.1; https://www.qgis.org). (b) Isotherms at 100 m below sea level (modified from Allis and Yusa^16^ and distribution of production rate (modified from Yusa^17^. Black squares (C3, BGRL): Location of gravity measurements and seismic observation, (C1): Reference point for gravity monitoring. Dashed black rectangle: Area analyzed for InSAR time-series displacement (Fig. 3).

However, there are no examples of the simultaneous use of the methods. Therefore, comprehensive reservoir monitoring was conducted in the present study based on InSAR, gravity, and PII in the Beppu geothermal area, which has the largest hot spring discharge and highest average temperature in Japan (Fig. 2). The geothermal resources in Beppu have been used as public baths (hot springs) for many years. The amount of hot spring water collected from 1949 to 1987 varied between 431 and 656 kg/s^14^, and geothermal use is still active today. In a geothermal power plant, the fluid used for power generation is injected into the ground; however, there are usually no injection wells in hot-spring projects because Japanese law does not state installation of injection wells in hot springs^15^. Wastewater is treated and discharged into sewage systems, and excess hot-spring water is discharged into rivers or the sea. Small-scale binary power plants have also been in operation in the Beppu area since 2014. When the fluid emerges from the subsurface, the geothermal reservoir is typically recharged by groundwater flow from the surrounding region. However, if production exceeds recharge capacity, fluid replenishment will not occur, and production would not be maintained. Therefore, monitoring surface and subsurface change is vital for sustainable geothermal resource utilization.

In the present study, ALOS-2 data were used for InSAR analysis to obtain time-series land surface deformation characteristics. Gravity measurements were conducted, and seismometers were installed at the C3 and BGRL sites in October 2021; C3 is in the Kannawa area, where geothermal activity and resource exploitation are particularly high. BGRL is located on the southern side of a fan-shaped area, and hot spring water flow is observed at several depths, although there is no significant geothermal sign at the surface (Fig. 2).

## Results and interpretation

### Synthetic aperture radar

**Fig. 3:**
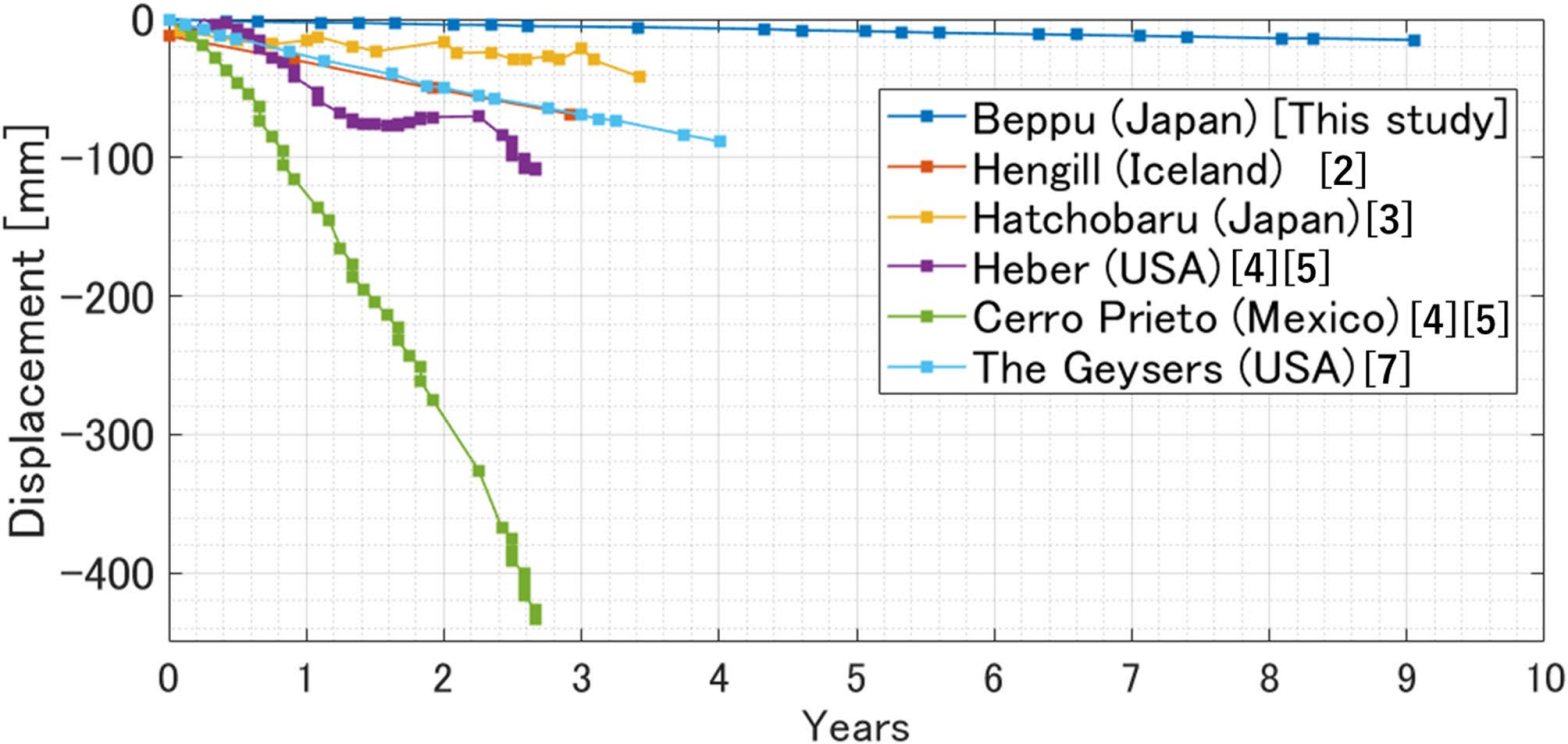
Time-series displacement obtained from InSAR in geothermal areas. Comparison of displacement scale across different geothermal fields, including Hengill (Iceland; 2018–2021)^2^, Hatchobaru (Japan; 2007–2010)^3^, Heber (USA; 2015–2017), and Cerro Prieto (Mexico; 2015–2017)^4,5^, The Geysers (USA; 2007–2011)^7^ and Beppu C3 area (this study; 2014–2023). Digitized values are taken from the respective references.

**Fig. 4:**
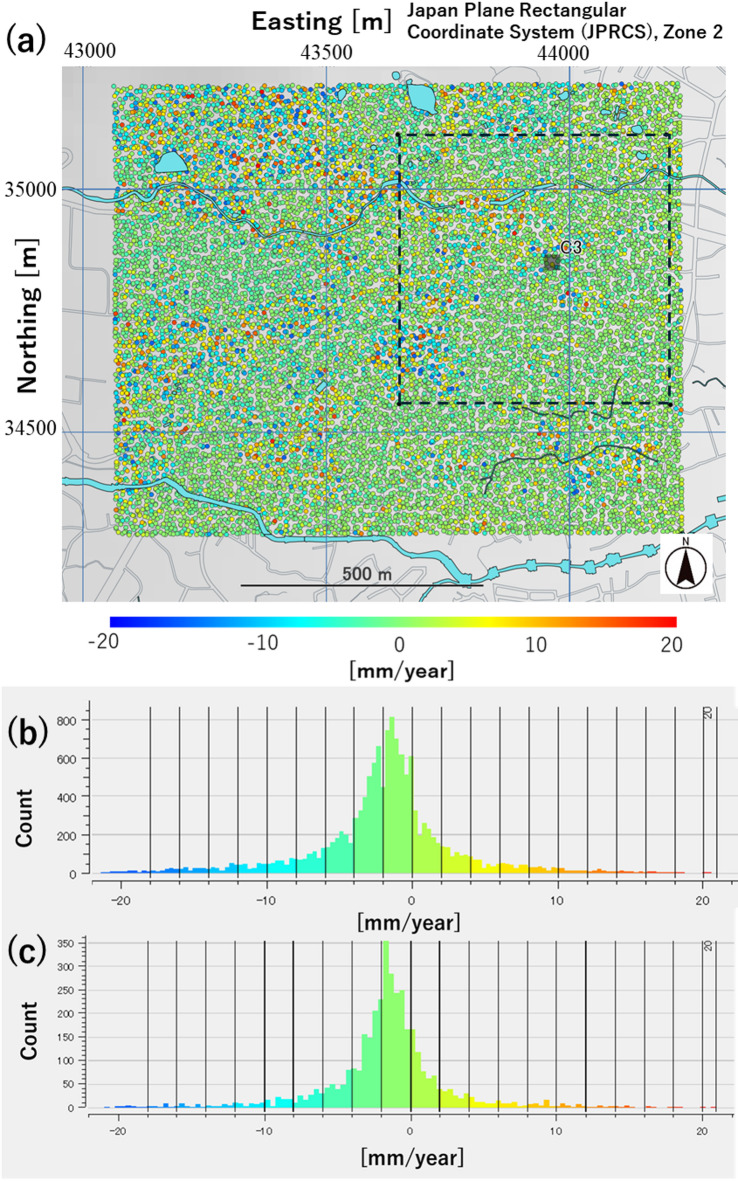
Average displacement rate [mm/year] derived from ALOS-2 L-band InSAR analysis around Beppu C3 area. (a) Spatial distribution of displacement rate (1 km mesh) between November 2014 and November 2023. The black rectangle indicates the area analyzed for InSAR time-series displacement (Fig. 3). (b) Histogram of average displacement rate based on 1 km mesh data. (c) Histogram of average displacement rate within the black rectangle.

InSAR analysis has previously identified subsidence associated with production in geothermal areas; more than 50 mm was observed over a three-year period at Hengill geothermal area, Iceland^2^, 40 mm was observed over three years around Hatchobaru geothermal power plant, Japan^3^, 100 mm was observed at Heber, USA over two and a half years, and 450 mm was observed over two and a half years at Cerro Prieto, Mexico^4,5^ (Fig. 3). The Geysers geothermal field exhibited 373 mm of maximum displacement over 7.25 years from 1992, corresponding to an average rate of approximately 50 mm/year^6^, and more recent InSAR observations also captured about 90 mm of subsidence over the four-year period beginning in 2007^7^. In contrast, settlement at the Beppu C3 area has been only 15 mm over the past decade (from November 2014 to November 2023), indicating that Beppu exhibits negligible subsidence despite active geothermal use (Fig. 3). Figure 4a shows the spatial distribution of average displacement rates during 2014–2023 based on the LIANA data used in this study (see Method 5.2.1), allowing the overall regional tendency to be captured. The corresponding histogram is presented in Fig. 4b. These results indicate that, throughout the period 2014–2023, only very small displacement rates—approximately − 2 to 0 mm/yr—occurred in and around the Beppu C3 area. The cumulative subsidence shown in Fig. 3 represents the average cumulative subsidence within the area enclosed by the black dashed box in Fig. 4a, and Fig. 4c provides the histogram of the average displacement rates for that same region. Figure 4c likewise confirms that deformation within this area is small overall. These findings demonstrate that ground deformation in the Beppu C3 region is extremely minor in spatial terms and does not reach the levels of subsidence reported in other geothermal fields.

Surface displacement is linked the pore pressure drop in the reservoir^18^. A decrease in pore pressure causes the reservoir to compress and the volume to decrease, leading to settlement. *K* (bulk modulus), *ν* (Poisson’s ratio), and reservoir depth also affect the magnitude of surface subsidence^18–20^; therefore, representative values reported in previous studies are summarized here (Poisson’s ratio represents an approximate range assuming the rock mass of the host formation). Hengill–Hellisheidi field is a geothermal system developed within basaltic formations, where the reservoir depth generally ranges from several hundred meters to about 2 km^2,21^. The bulk modulus *K* estimated from surface deformation has been reported to be approximately 1.3–6.4 GPa^19^. The Poisson’s ratio of typical basalt is on the order of 0.1–0.35^22^, and a value of 0.25 is adopted as an assumed parameter in subsurface modeling^23^. The Geysers is the world’s largest vapor dominated geothermal system, where high temperature zones (> 260 ℃) are distributed mostly below depths of ~ 1500 m^24^. Based on estimated volumetric strain and observed reservoir pressure changes, the bulk modulus has been constrained to K ≤ 4.6 GPa^25^. The Poisson’s ratio of the graywacke host rock is generally in the range of 0.1–0.2^22^, and values within 0.1–0.4 have been used as assumed parameters in previous studies^19^. The InSAR results for the Beppu area indicate no significant reduction in reservoir volume. In the Beppu geothermal field, where the shallow geological units are mainly composed of alluvial sediments and tuff breccias, the reservoir is located at depths of a few hundred meters, and temperatures show a convective profile below a depth of approximately 200 m near the C3 site^16^. Typical Poisson’s ratio values range from approximately 0.15 to 0.35 for sand and gravel, and from about 0.1 to 0.3 for tuff^22^. Despite this shallower reservoir compared with other geothermal fields, both the magnitude and rate of displacement are small. This may be attributed to the relatively high bulk modulus in Beppu (~ 7 GPa; Methods 5.3) and to the fact that, although the entire Beppu area produces roughly 50,000 tons/day, the production volume in the vicinity of the C3 site is comparatively low.

### Comparative observations of gravity and velocity

**Fig. 5:**
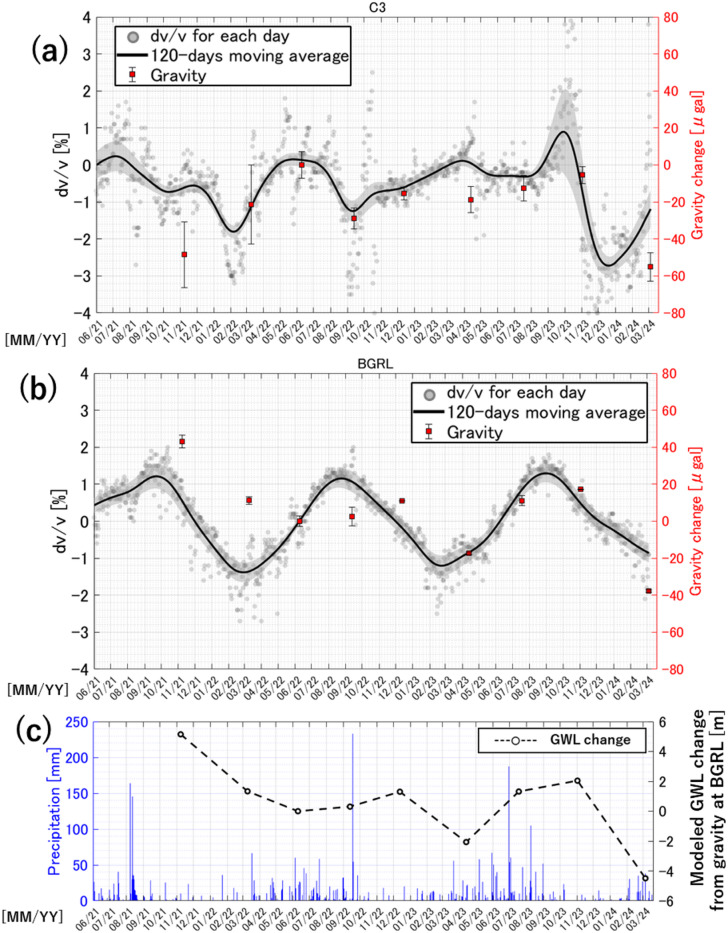
Gravity and velocity changes. (**a**) C3. Reference time was set as June 2022, when the gravity value at C3 was high and gas saturation was relatively low (close to liquid saturation) during the observation period. Red points with error bars: gravity changes; Gray points: daily dv/v estimates; Black line: 120‑day moving average of dv/v; Gray band: 120‑day moving average of dv/v uncertainties (Methods 5.2.3). (b) BGRL. Reference time was set to June 2022, the same as C3. (**c**) Precipitation of AMEDAS OITA (location is shown in Fig. 2a) and estimated shallow groundwater-level changes at BGRL derived from the observed gravity variations (Methods 5.2.2).

Comparisons of the gravity and velocity changes since 2021 are shown in Fig. 5. Gravity and velocity varied over relatively short periods, although no significant subsidence was observed. Gravity and velocity at C3 changed irregularly (Fig. 5a), with no periodicity, where geothermal manifestation and usage were particularly active. Conversely, seasonal variations were observed at the BGRL stations (Fig. 5b), where there was no discernible geothermal activity. At BGRL, gravity exhibits a cycle with higher values typically in late autumn to early winter (November) and lower values toward spring (March–April); however, the seasonal cycle and amplitude vary slightly from year to year. The gravity change in 2022 shows a small amplitude, decreasing toward June and then increasing again toward December. The results of comprehensive monitoring using InSAR, gravity, and PII suggest that reservoir compaction is not occurring; however, events that contribute to gravity and velocity changes are occurring in the subsurface.

## Discussion

In general, in long‑established hot‑spring regions, public bath use has historically taken precedence, and systematic monitoring has not been actively conducted. However, sustained utilization of geothermal resources in the future will require continuous assessment of subsurface conditions, and the use of non‑invasive monitoring approaches is increasingly expected. In this context, the present study applied non-invasive methods to examine what kinds of variations can be obtained from the data. Therefore, the interpretations in this study are constrained by data availability. Continuous time‑series of production rate and groundwater‑level observations were not available in the Beppu hot‑spring area, limiting the ability to separate the causal contributions of production‑induced effects and natural recharge. Consequently, the interpretations rely on indirect observation.

### Factors influencing short-term variation

Comparative observations over two and a half years revealed some short-term changes in the subsurface during periods when no significant subsidence was observed (Figs. 3 and 5). The negligible subsidence implies that short-term variations in gravity are mainly caused by changes in subsurface saturation. BGRL, which shows periodic changes, is likely linked to seasonal water level changes in unconfined aquifers. The irregular variations at C3 are thought to be influenced by changes in reservoir saturation. The velocity changes can be caused by the following factors: (1) changes in crustal stress under earthquakes^26^, (2) damage to the surface layer and cracks in the subsurface associated with earthquakes^27^, (3) diffusion of fluid deep into open cracks^28^, (4) rigidity change with saturation change caused by rainfall infiltration^13,29^, (5) change in bulk modulus caused by geothermal fluid production^2^. At C3, dv/v decreased from June to September 2022, matching a gravity decrease over the same period. In 2023, dv/v declined from October to December. While gravity is discussed on a campaign‑month basis and no campaign was conducted in October 2023, subsequent measurements indicate a downward trend from November 2023 to March 2024 (Fig. 5a). No large earthquakes occurred in Beppu near the period. Since similar velocity trends are not observed in C3 and BGRL in the same Beppu City, the velocity changes are not considered to be caused by stress changes or damage due to earthquakes.

**Fig. 6:**
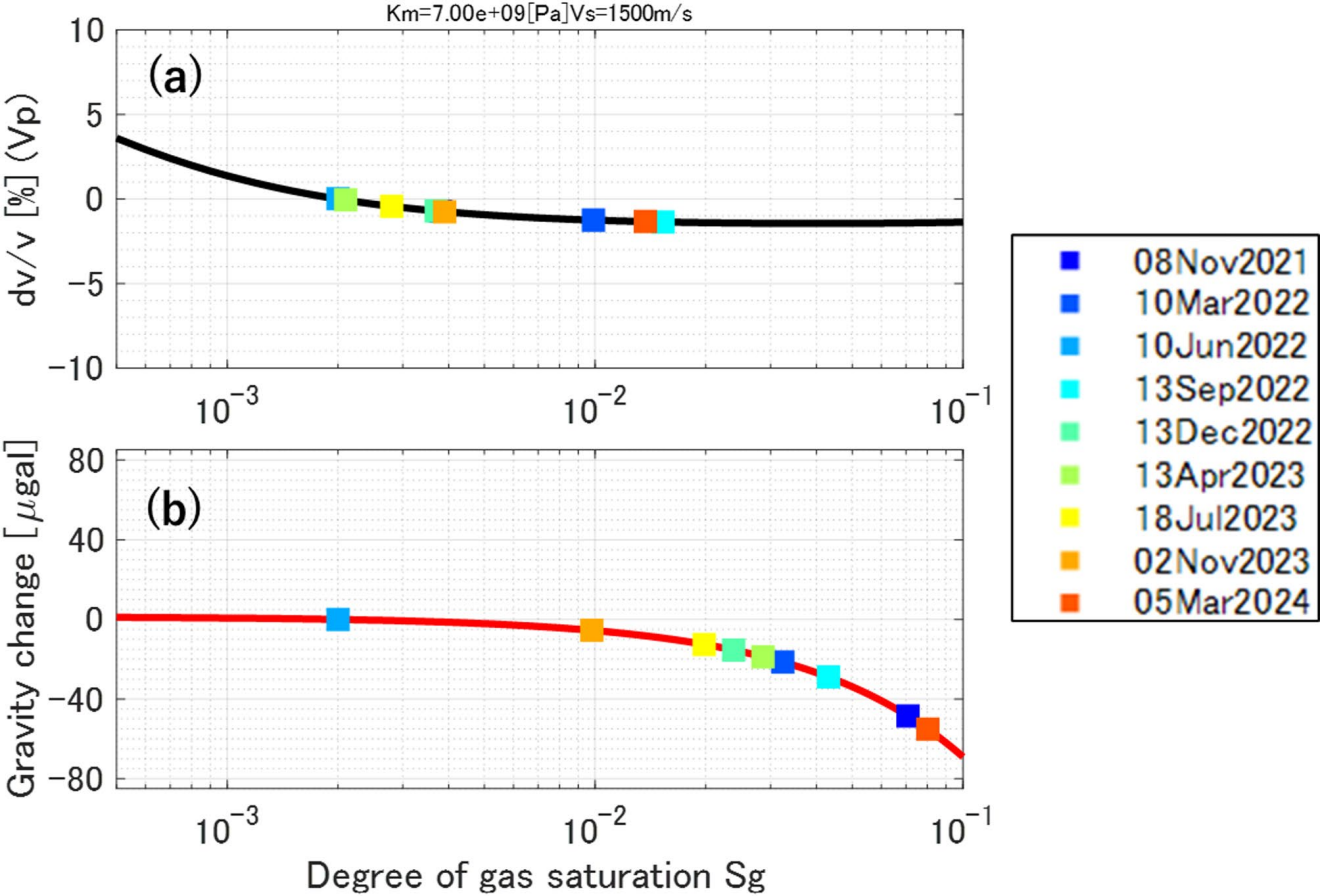
Theoretical velocity and gravity changes against gas saturation based on assumptions of reservoir size and physical properties. The color plots are the observed dv/v and gravity changes at C3.

**Fig. 7:**
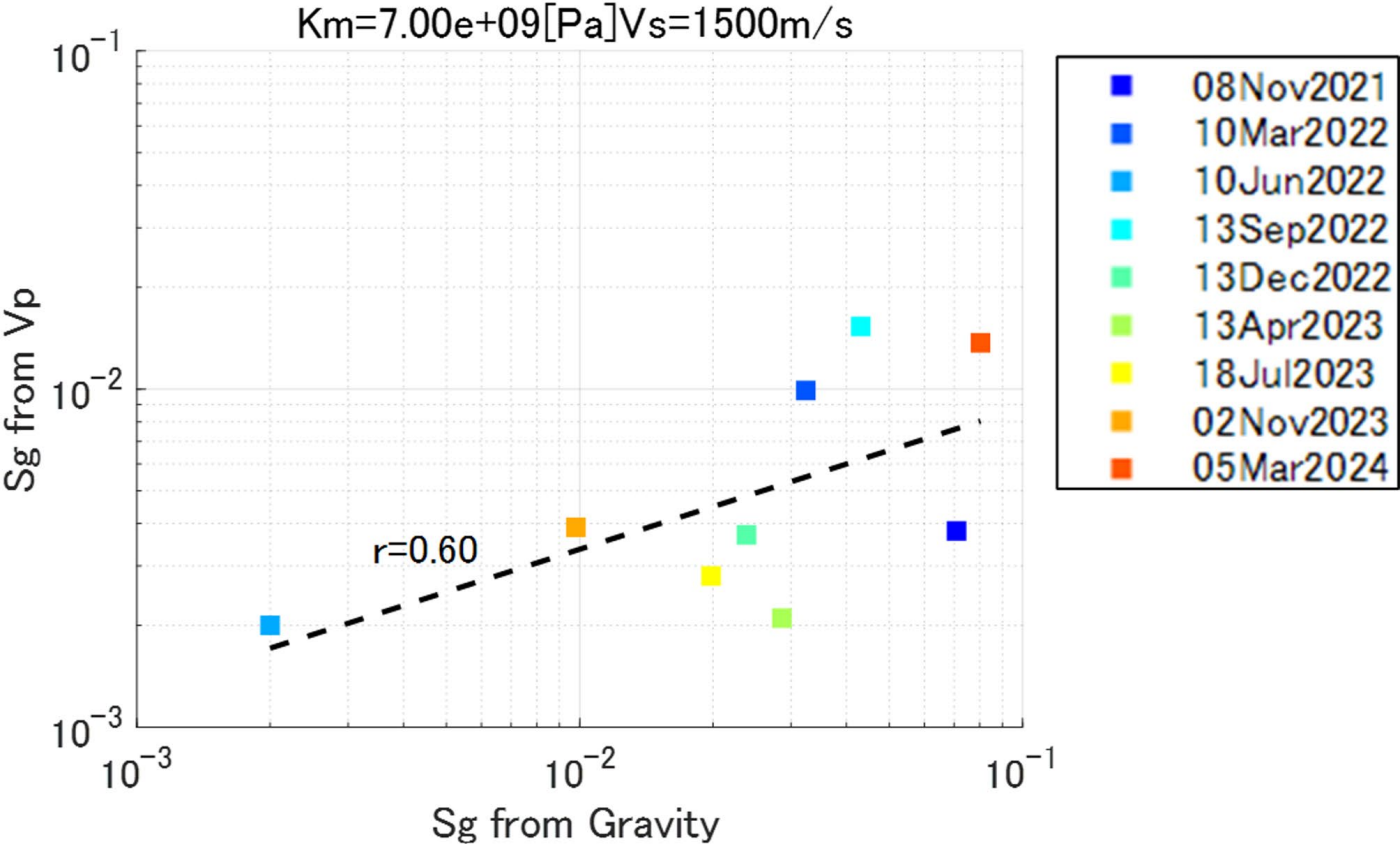
Gas saturation estimated from observed dv/v (Vp) and gravity change.

Shear modulus is not affected by changes in saturation. Conversely, as gas saturation increases, Vp decreases because bulk modulus decreases owing to substitution of water by air in the pores^30^. The results of the present study show that velocity exhibits a similar trend to gravity, suggesting that velocity is also influenced, at least to some extent, by changes in gas saturation. Here, the observed velocity change is considered to be a change in Vp, and velocity changes and gravity changes corresponding to gas saturation were calculated based on rock physics model^31,32^ (Fig. 6). The reservoir area (1 km^2^) and depth (200–350 m) used for gravity calculations were set with reference to the size of the Kannawa area and depth of hot-spring wells, and the physical properties used in calculation of velocity changes were referenced to literature values^33,34^ (Methods 5.3). In simplifying the subsurface structure, several assumptions must be introduced; among them, bulk modulus of the matrix has a particularly strong influence on seismic velocity. Therefore, in method 5.3, theoretical curves were computed for multiple values of the matrix bulk modulus, and Fig. 6 presents the theoretical curve that shows the highest consistency with the observed values. Based on the theoretical calculation, gas saturation was estimated based on observed velocity changes and gravity changes (Fig. 7). Although the gas saturation estimated from each method did not match entirely, a correlation of *r* = 0.6 was obtained, and the similar observed velocity and gravity trends are considered to be influenced by changes in gas saturation. The above are very simplified considerations of the underground structure based on assumptions; however, gas saturation is expected to vary between 0 and several percentage points during the observation period.

The rapid decrease in velocity at C3 may indicate that the fluid in the reservoir began to vaporize. After the decrease in velocity (and gravity), it tended to increase, suggesting that fluid recharging from the surrounding area caused the pressure to increase again and gas saturation to decrease.

Although continuous groundwater-level observations are unavailable, past measurements at BGRL have documented water levels in the unconfined aquifer, and Nishijima et al.^35^ have shown that gravity and groundwater-level variations at this site fluctuate synchronously. Based on this knowledge, we estimated shallow groundwater-level changes for the study period from the observed gravity variations using theoretical conversion (Methods 5.2.2), and the results are presented in Fig. 5c. Consistent with long-term observations in the Beppu area—where the unconfined groundwater level typically fluctuates between ~ 40 and 50 m a.s.l., peaking around October–November and reaching a minimum around April–May^36^—our inferred water level at BGRL exhibits maxima in November 2021, December 2022, and November 2023, and minima in June 2022, April 2023, and March 2024. Taken together with the seasonality of gravity at BGRL (Fig. 5b) and the absence of nearby production activities, we interpret the relatively smooth, continuous dv/v variations at BGRL as seasonal rock-property changes driven by rainfall infiltration and groundwater-level fluctuations, rather than phase-change processes within a confined reservoir. The fact that dv/v and water-level changes are not perfectly antiphase likely reflects the combined influence of effective-stress changes and drainage conditions (permeability, storage coefficient, and the thickness of the vadose zone), which may together influence the timing and magnitude of stiffness reductions as saturation increases.

### Insights from the application of non-invasive monitoring

**Fig. 8:**
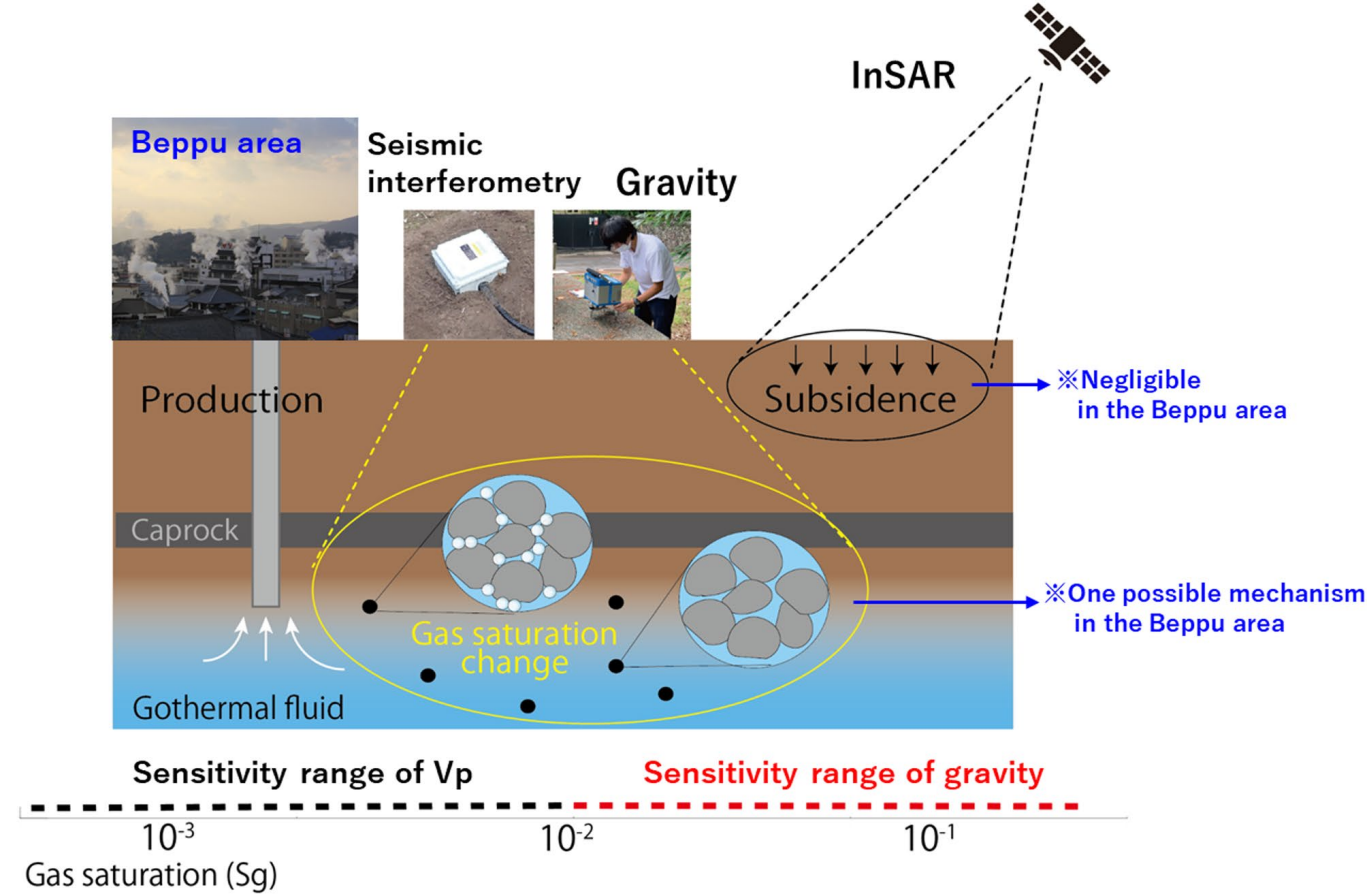
One possible conceptual mechanism of subsurface changes in the Beppu area.

Non-invasive monitoring methods are expected to provide indirect indications of possible subsurface changes (Fig. 1). Figure 8 extends the conceptual framework summarized in Fig. 1 by incorporating the observational evidence from InSAR and one possible scenario inferred from the gravity and velocity variations obtained in this study.

The magnitude and spatial pattern of subsidence in geothermal areas are influenced by the mechanical properties of the reservoir, including its effective bulk modulus, Poisson’s ratio, and depth. In general, deeper reservoirs produce smaller peak subsidence but affect a wider area. Pore‑pressure decline can also directly reduce reservoir volume, thereby causing surface subsidence^18^. In the Beppu geothermal field, no significant subsidence has been observed, suggesting that large‑scale pressure depletion has not occurred.

In contrast, gravity and seismic velocity exhibited short‑period variations even during periods when InSAR detected no significant subsidence. Although multiple processes may contribute to these variations, we showed that, if several percent of gas‑saturation change had occurred in the reservoir in association with production activities, the resulting changes in density and elastic properties could give rise to the gravity and velocity changes. While this represents only one possible explanation, the presence of short‑period variations in both gravity and velocity suggests that these non‑invasive measurements may have the potential to complementarily capture early‑stage changes in production areas.

As a direction for future work, applying this multi‑method monitoring framework to geothermal power‑plant fields where production and groundwater‑level data are continuously recorded would enable a more quantitative evaluation of the relative contributions of production and recharge, and facilitate more rigorous causal separation.

This conceptual figure is based on the general processes summarized in Fig. 1 and illustrates one possible subsurface evolution inferred from the theoretical relationships between gas saturation, velocity, and gravity based on this study (Fig. 6).

## Limitations

### Lack of production rate time series and in situ GWL

In the Beppu hot-spring area, continuous time-series data for production rate and groundwater level were not available. As a result, it was not possible to rigorously separate the causal contributions of production-induced changes from those driven by natural recharge. Consequently, the interpretations in this study are based on the results obtained from non-invasive monitoring methods. For this reason, our focus is not on identifying direct causal relationships but rather on presenting what kinds of subsurface changes could potentially be observed.

### Potential cancellation of simultaneously occurring processes

When an increase in reservoir gas saturation (Sg) lowers both Δg and dv/v, and processes that increase gravity or stiffness occur simultaneously, these effects may cancel each other out, making detection difficult; such processes include pronounced subsidence (Δg↑), seasonal rises in shallow groundwater level (Δg↑), compression‑related stiffening (dv/v↑), and stiffness enhancement associated with seasonal lowering of shallow groundwater level (dv/v↑).

## Conclusion

This paper presents the findings of a non-invasive monitoring survey using InSAR, gravity, and PII of the reservoir in the Beppu hot spring area. Comparative observations showed that gravity and velocity varied over short periods at the high-geothermal activity and geothermal resource exploitation site, even though no significant subsidence was observed over the same period. Although multiple processes may contribute to these variations, we demonstrated that, as one possible explanation, if several percent of gas-saturation changes had occurred in association with production activities, the resulting changes in density and elastic properties could have given rise to the observed gravity and velocity changes.

Attempts to capture short‑term subsurface variations through gravity and velocity monitoring may contribute to assessing the risk of overextraction, similarly to long‑term InSAR deformation time‑series analyses. Such comprehensive monitoring promotes operator understanding of reservoirs in production management and leads to social acceptance.

## Methods

### Study area

**Fig. 9:**
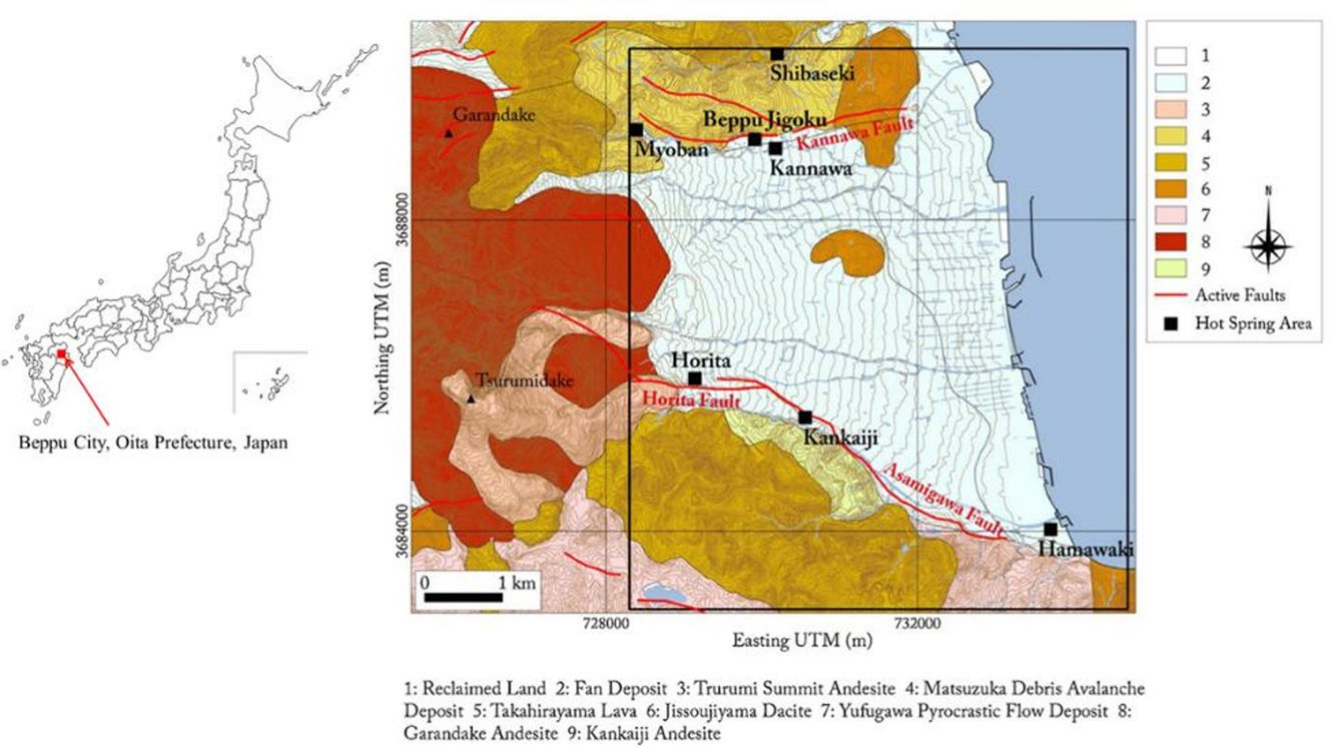
Geology map of Beppu geothermal field^35^. (modified from Geological Survey of Japan and the National Institute of Advanced Industrial Science and Technology [AIST]^37^.

The Beppu area is one of the largest hot spring areas in Japan, with more than 2,000 sources. Geothermal energy in the area is harnessed primarily from hot springs. Beppu area geology is characterized by a Quaternary volcanic group consisting of Tsurumidake (Mt. Tsurumi) and Garandake (Mt. Garan), located at the northeastern end of the Beppu-Shimabara rift, and an alluvial fan area extending from the foot of the mountains to Beppu Bay^37^ (Fig. 9). The Kannawa and Asamigawa Faults are located on the northern and southern margins of the fan, respectively. Tuff, lava, and pyroxene andesites are deposited in the lower part of the fan deposits, which are several hundred meters thick^38^. Compositional analysis of the hot spring water revealed that fluid flows in multiple pathways from the upstream side, with Mt. Tsurumi and Mt. Garan located downstream of the coast^39,40^ (Fig. 2). The flow paths are largely divided into two parts on the north and south sides of the Beppu area, flowing along the Kannawa Fault on the north margin and the Asamigawa Fault on the south side. Hot springs are distributed mainly in the fan area. In particular, hot springs in the Myoban, Kannawa, Shibaseki, Kamegawa, Horita, Kankaiji, Beppu, and Hamawaki areas are representative and are called Beppu Hattou.

### Methods of monitoring geothermal reservoir

One method of monitoring geothermal reservoir (hot spring aquifer) is measurements using wellbores. To monitor wellbore temperature and pressure over a long period, a sensor that can withstand high temperature and pressure is required (e.g., optical fiber sensors). However, from a cost perspective, measurement data are basically taken at the wellhead in hot spring areas. In many cases, data cannot always be obtained owing to silica scaling or other problems in wellbores. Therefore, monitoring the subsurface without using wells is an effective approach. In addition, from a long-term monitoring perspective, low-manpower and low-cost methods are preferred. Therefore, this study focused on repeat gravity measurements, PII, and InSAR.

#### InSAR

Ground deformation data was obtained from the LIANA service^41^ (developed by SKY Perfect JSAT Corporation, ZENRIN Co., Ltd., and Nippon Koei Co., Ltd.), which provides InSAR-based surface displacement data derived from Differential InSAR (D-InSAR) analysis of ALOS-2 PALSAR-2 images. The processing follows a standard D-InSAR workflow, including co-registration, interferogram generation, removal of topographic phase using a DEM, phase unwrapping, and conversion to LOS displacement, as described by the Geospatial Information Authority of Japan (GSI)^42^. LIANA provides displacement data on a 1 km mesh (Fig. 4a).

The 22 images used for the analysis were acquired between November 2014 and November 2023 (Table 1), all in HH polarization, right-looking mode, on ascending orbits, and provided as Level 1.1 products representing displacement in the satellite line-of-sight (LOS) direction. The average displacement rate map is shown in Fig. 4a, and its histogram is presented in Fig. 4b and c, indicating that the overall displacement rates are relatively small. The time-series of displacement was calculated within an approximately 500 m square area surrounding C3 (Fig. 3).

**Table 1:** List of ALOS-2 images analyzed (Acquisition date of master image is 7 Nov, 2014).

Pair no	Acquisition date slave image	Time (JST)
1	10 Apr, 2015	0:11:47
2	3 Jul, 2015	0:11:45
3	18 Dec, 2015	0:11:44
4	25 Mar, 2016	0:11:43
5	1 Jul, 2016	0:11:41
6	2 Dec, 2016	0:11:42
7	10 Mar, 2017	0:11:40
8	16 Jun, 2017	0:11:38
9	6 Apr, 2018	0:11:40
10	8 Mar, 2019	0:11:42
11	14 Jun, 2019	0:11:39
12	29 Nov, 2019	0:11:41
13	6 Mar, 2020	0:11:43
14	12 Jun, 2020	0:11:40
15	5 Mar, 2021	0:11:44
16	11 Jun, 2021	0:11:41
17	26 Nov, 2021	0:11:43
18	1 Apr, 2022	0:11:43
19	9 Dec, 2022	0:11:44
20	3 Mar, 2023	0:11:44
21	24 Nov, 2023	0:11:42

#### Gravity measurements

**Fig. 10:**
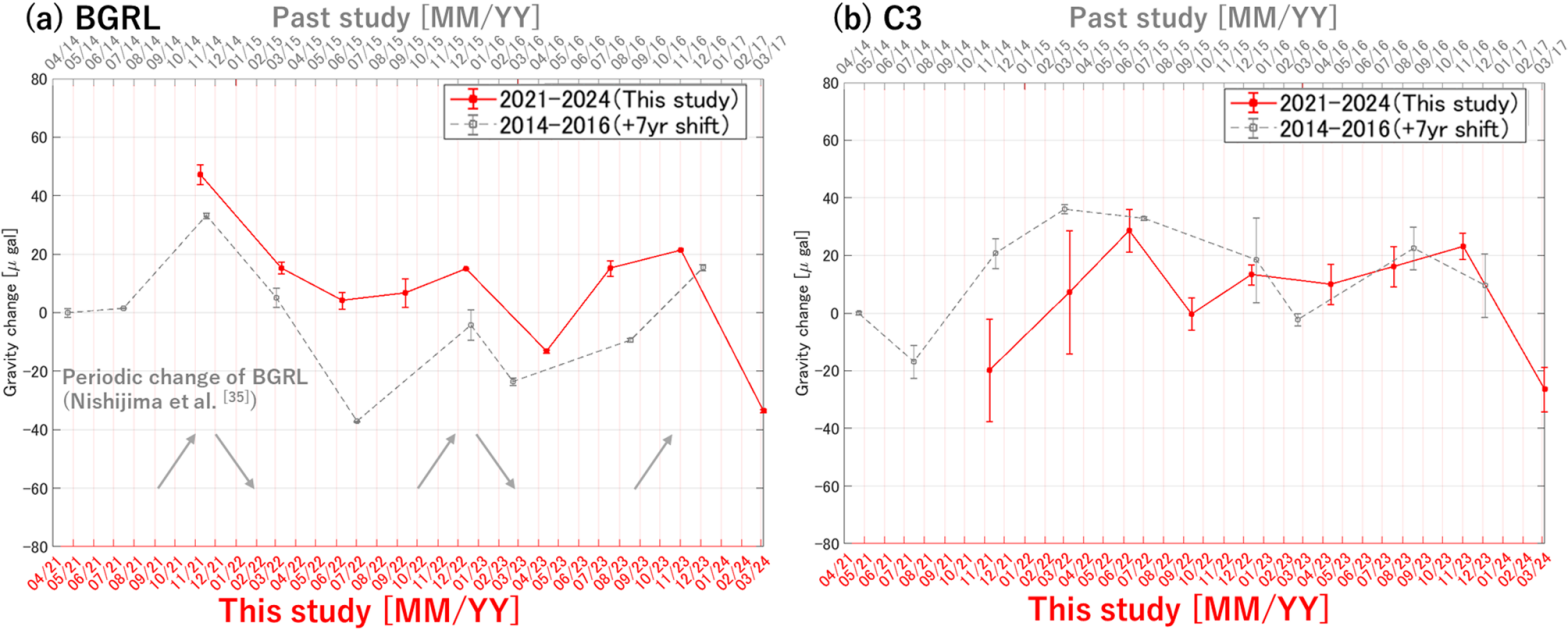
Gravity changes at both stations combine data from the previous study (Nishijima et al.^35^; 2014–2016) and this study (2021–2024; referenced to April 2014). The past‑study data are shifted by seven years to align the months (seasonal phase) with the observation period of this study. Error bars represent differences between outward and return drift‑corrected values, with averages shown as squares. (a) BGRL; (b) C3.

In the present study, gravity measurements were conducted since 2021 using relative gravimeters. Closed loops were formed with the measuring points, and round-trip measurements were performed to effectively detect and analyze gravity changes. In each of the outbound and return measurements, gravity values were measured multiple times until they settled within 10 µgal at each measurement point. After applying tide, machine height, and drift corrections to the obtained data, the relative gravity differences from the reference point were calculated. The reference point was C1, which was the closest to the sea and was considered to have the smallest change in groundwater saturation. Figure 10 shows gravity trends over time at C3 and BGRL, including the period covered by previous studies^35^. To observe the long-term trends, the time reference was April 2014, the start of monitoring by Nishijima et al.^35^, and the value at each time point is shown as the gravity change from April 2014, the reference time. The gravity at C3 changed irregularly between − 20 µgal and 40 µgal. The periodic BGRL trend since 2014 has been shown.

##### Shallow groundwater-level change estimated from gravity

Shallow groundwater-level changes can be estimated from gravity variations using Eq. (1)^11^, assuming an infinite slab aquifer:

1 $$\Delta h=\frac{{\Delta g}}{{2\pi G{\rho _W}n}}$$

where $\Delta h$, $\Delta g$, *G*, ${\rho _w}$, and *n* denote the change in groundwater level (m), the observed gravity change (µgal (= 10^− 8^m/s^2^)), gravitational constant (6.674 × 10^− 11^ m^3^/kg/s^2^), the density of water (assumed 1000 kg/m^3^) and the porosity (assumed 0.20), respectively.

#### Passive image interferometry

In an application in areas with geothermal power plants, Sánchez-Pastor et al.^2^ captured velocity drops of about 1–2%/year from 2018 to 2021 near the Hetlisheidi geothermal power plant in the Hengill geothermal region, Iceland. Reservoir simulations using iTOUGH2^43^ in the study area showed a marked decrease in pressure and increase in gas saturation associated with production activities. The velocity decrease from the ACF was interpreted as a decrease in Vp (bulk modulus) owing to vaporization in the reservoir.

##### Data acquisition and processing

**Fig. 11:**
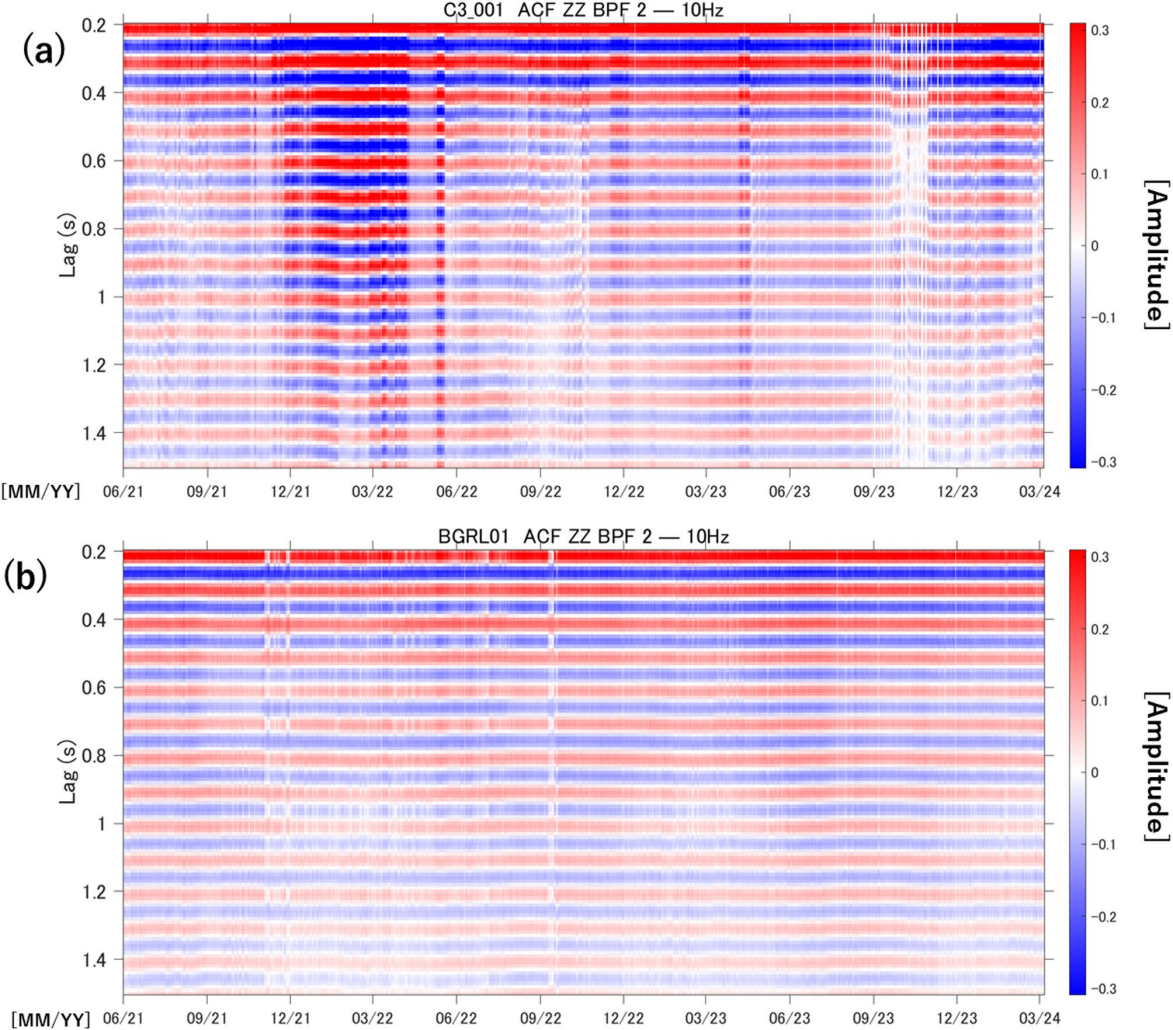
Temporal variation in the vertical component of autocorrelation functions (ACFs) (a) C3 (b) BGRL. The frequency band was 2–10 Hz. Warmer colors denote higher ACF amplitude.

Seismometers were installed at C3 and BGRL for microtremor observations in the present study. The sampling frequency was 100 Hz. BGRL replaced the seismograph in September 2022 because of suspected seismograph failure due to the lack of aligned spectra below 3 Hz. Vertical‑component signals were first band‑pass filtered, after which one‑bit normalization was applied to minimize the influence of transient, non‑noise signals. Autocorrelation functions (ACFs) were computed for each 1‑hour segment of the continuous record, and the 24 hourly ACFs were then averaged to produce a single 1‑day ACF (Fig. 11). The velocity change was determined from the phase of the obtained ACFs using the stretching interpolation method^13^.

Because dv/v is sensitive to the choice of analysis parameters —specifically the selected frequency band and lag‑time window—some arbitrariness is generally unavoidable in choosing them. In this study, gravity changes were used as an independent constraint. Because gravity reflects subsurface mass (saturation) variations, gravity and velocity are expected to share the same trend when phase changes occur in response to production in geothermal areas (i.e., an increase in gas saturation leads to decreases in both gravity and P‑wave velocity). Accordingly, we selected the dv/v analysis parameters—namely the frequency band (2–10 Hz) and the lag‑time window (0.6–1.0 s)—such that the dv/v trend at C3 follows the gravity‑inferred trend observed at this production-active site. For BGRL, although it is not located in a production area, we applied the same parameter settings for comparison.

##### Uncertainty of the dv/v

**Fig. 12:**
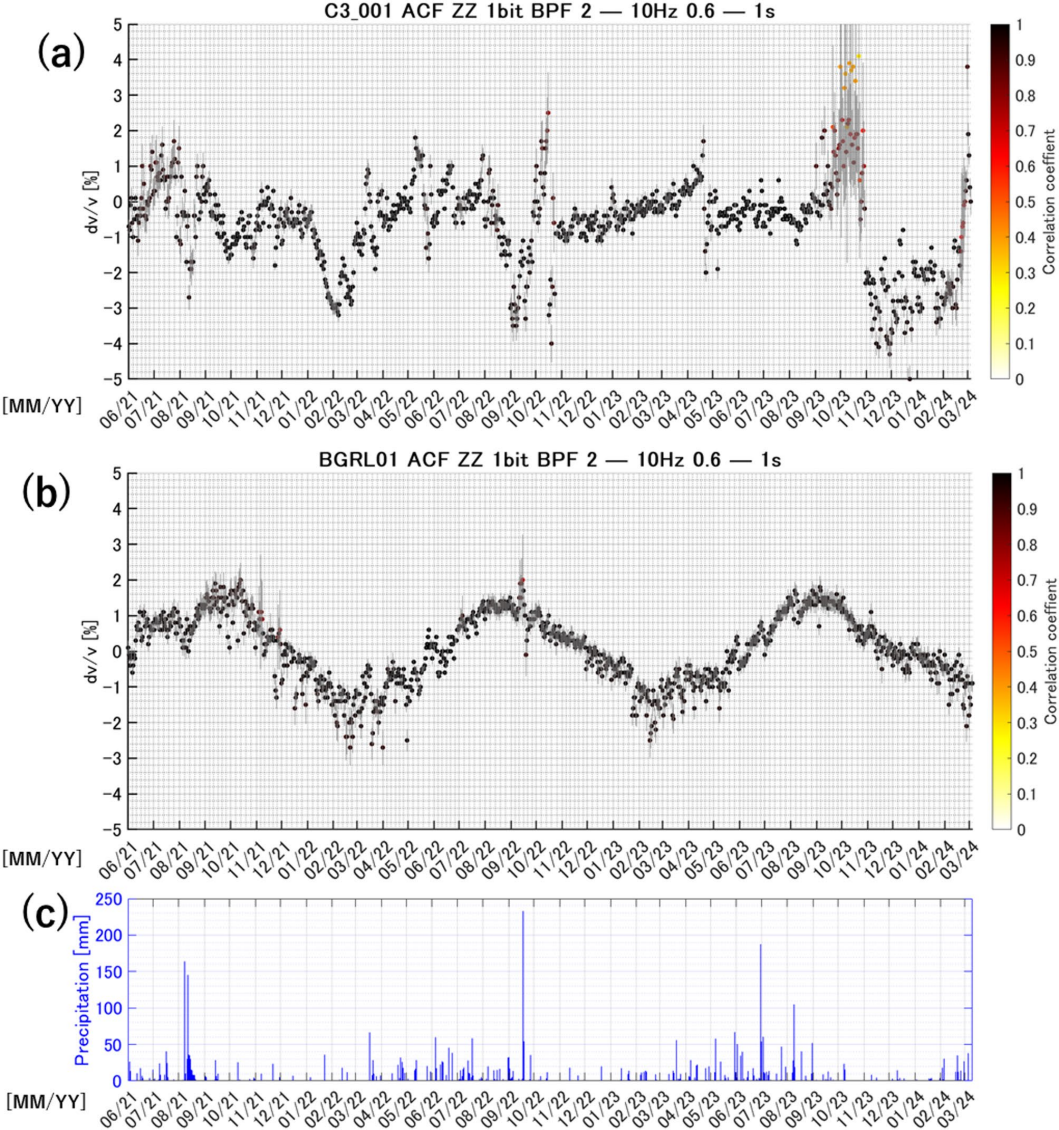
Temporal variations in relative velocity change (dv/v). (a) C3; (b) BGRL. dv/v was estimated using the stretching method with a 0.6–1.0 s lag window. The gray error bars represent the uncertainties calculated using Eq. (2). The color of the circles indicates the stretching correlation coefficients. (c) Daily precipitation recorded at AMEDAS OITA.

To evaluate the uncertainty of dv/v estimates, Weaver et al.^44^ proposed an analytical expression that predicts the level of stretching‑coefficient fluctuations expected even when the seismic velocity of the medium remains unchanged (Eq. 2). Following their approach, we evaluated the uncertainty in this study as well. This formulation provides an intuitive estimate of apparent dv/v variability arising solely from changes in noise sources or other non‑structural factors, considering both the coherence and the analysis parameters specified in the stretching method^44,45^.

2 $$err=\frac{{\sqrt {1 - {C^2}} }}{{2C}}\sqrt {\frac{{6\sqrt {\pi /2} T}}{{\omega _{c}^{2}\left( {t_{2}^{3} - t_{1}^{3}} \right)}}}$$

where *C*, *T*, ${\omega _C}$, ${t_1}$ and ${t_2}$ denote the maximum coherence of the stretching correlation, the inverse of the frequency bandwidth, the central angular frequency of the passband, and the start and end times of the lag window used in the stretching technique, respectively. Specifically, the uncertainty increases under conditions of low coherence, narrow bandwidth, low central angular frequency, and short lag windows. Daily dv/v errors were visualized using error bars, while the stretching correlation coefficients were indicated by color coding, as shown in Fig. 12.

In Fig. 5, to enable comparison with the gravity time resolution, a 120‑day moving average of the dv/v uncertainties was also computed and plotted as a gray band. Although C3 exhibits larger uncertainties during some intervals, the amplitude of the observed dv/v variations generally exceeds the estimated errors at both stations, indicating that the main temporal features are significant (Fig. 12).

### Vp and gravity change versus gas saturation

Vp varies greatly with the inclusion of liquids and gases because the bulk modulus μ is affected by water and air, unlike the shear modulus µ.

3 $${V_p}=\sqrt {\frac{{K+\frac{4}{3}\mu }}{\rho }}$$

**Fig. 13:**
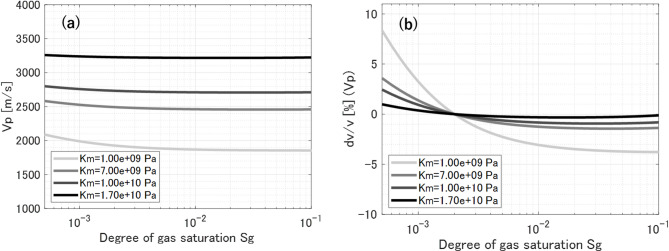
Vp and dv/v (Vp change) against gas saturation. ${K_m}$ was considered for several values up to $1.7 \times {10^{10}}\left[ {Pa} \right]$. The velocity changes were calculated with reference values at gas saturation of 2 × 10^− 3^.

The bulk modulus *K* of the ground considering two-phase system can be expressed by the following equations^31,32^:

4 $$K={K_m}+{a^2}M$$

5 $$a=1 - {K_m}/{K_s}$$

6 $$M={K_s}{\left[ {a+\frac{n}{{{K_f}}}\left( {{K_s} - {K_f}} \right)} \right]^{ - 1}}$$

7 $${K_f}=\frac{1}{{\frac{{{S_g}}}{{{K_{f1}}}}+\frac{{1 - {S_g}}}{{{K_{f2}}}}}}$$

where ${K_{f1}}$, ${K_{f2}}$, ${K_m}$, and ${K_s}$ are bulk moduli of the gas, liquid, matrix, and soil particles, respectively. *n* is the porosity. The density ρ of the ground considering a two-phase system is obtained from:

8 $$\rho =\left( {1 - n} \right){\rho _S}+n[{S_g}{\rho _{f1}}+\left( {1 - {S_g}} \right){\rho _{f2}}]$$

where ${\rho _S}$ is soil particle density, ${\rho _{f1}}$is gas density, and ${\rho _{f2}}$ is liquid density. Based on the results of an array survey, Vs was set to 1500 m/s (shear modulus was fixed), and Vp with gas saturation was obtained under the following assumptions: ${K_{f1}}=3.03 \times {10^5}\left[ {Pa} \right]$^33^, ${K_{f2}}=2.31 \times {10^9}\left[ {Pa} \right]$^33^,${\rho _{f1}}=0\left[ {kg/{m^3}} \right]$^33^,${\rho _{f2}}=1.0 \times {10^3}\left[ {kg/{m^3}} \right]$^33^, ${K_s}=3.70 \times {10^{10}}\left[ {Pa} \right]$^34^, ${\rho _S}=2.65 \times {10^3}\left[ {kg/{m^3}} \right]$^33^. The porosity was set to *n* = 0.2, as in the gravity calculation. ${K_m}$ was considered for several values up to $1.7 \times {10^{10}}\left[ {Pa} \right]$^34^ (Fig. 13a). For the theoretical velocity change to be within ± a few percent of the observed value, ${K_m}$ was set to $7 \times {10^9}$, and a reference gas saturation for calculating the velocity change was set to $2 \times {10^{ - 3}}$ (Figs. 6a and 13b).

The theoretical equation presented in Okabe^46^ was used to calculate gravity change with gas saturation (Fig. 6b). The reservoir was assumed to be 1 km^2^, including the entire Kannawa area, and to have a thickness of 150 m. The depth of the upper part of the reservoir was assumed to be 200 m, referring to the depth of the hot-spring wells. Porosity was set to 0.2. Based on the assumptions, the gas saturation was estimated using the velocity changes and gravity changes obtained from observations (Fig. 7).

## Data Availability

The data of this study are available from the corresponding author on reasonable request.
